# Efficacy and biological safety of lopinavir/ritonavir based anti-retroviral therapy in HIV-1-infected patients: a meta-analysis of randomized controlled trials

**DOI:** 10.1038/srep08528

**Published:** 2015-02-23

**Authors:** Xiaojie Huang, Yuanlong Xu, Qiuying Yang, Jieqing Chen, Tong Zhang, Zaicun Li, Caiping Guo, Hui Chen, Hao Wu, Ning Li

**Affiliations:** 1 Center for Infectious Diseases, Beijing You'an Hospital, Capital Medical University, Beijing 100069, China; 2 Guangxi Longtan Hospital, Liuzhou, Guangxi 545005, China; 3 School of Biomedical Engineering, Capital Medical University, Beijing 100069, China; 4 Beijing Key Laboratory of Fundamental Research on Biomechanics in Clinical Application, Capital Medical University, Beijing 100069, China

## Abstract

Lopinavir/ritonavir (LPV/r) is the first ritonavir-boosted protease-inhibitor used in second-line anti-retroviral treatment (ART) in resource-limited regions. To evaluate the efficacy and safety outcomes of LPV/r in treatment-naïve and -experienced HIV-infected adults and pregnant women, we performed a meta-analysis of randomized controlled trials. Ten cohorts from 8 articles involving 2,584 ART-naïve patients, 5 cohorts from 4 articles involving 1,124 ART-experienced patients, and 8 cohorts from 7 articles involving 2,191 pregnant women were selected for the meta-analyses. For ART-naïve patients, the virologic response rate (72.3%) of LPV/r combined with tenofovir (TDF) plus lamivudine/emtricitabine (3TC/FTC) arms was significantly greater than that of LPV/r plus non-TDF-FTC arms (65.5%, p = 0.047). For ART-experienced patients, the use of LPV/r revealed a 55.7% probability of virologic success. The incidence of abnormal total cholesterol (6.9%) for ART-experienced patients was significantly lower than that for ART-naïve patients (13.1%, p < 0.001). The use of LPV/r in pregnant women revealed a mother-to-child transmission (MTCT) rate of 1.1%, preterm birth rate of 13.2%, and low birth weight rate of 16.2%. Our meta-analysis indicated that LPV/r was an efficacious regimen for ART-naïve patients and was more tolerable for ART-experienced patients. LPV/r also displayed a significant effect in preventing MTCT.

Antiretroviral therapy (ART) is a kind of treatment using anti-HIV drugs for people who infect with human immunodeficiency virus (HIV). Continuous improvements in ART have transformed HIV infection from a debilitating fatal disease into a chronic treatable disease^1^,^2^. In spite of the fact that majority of acquired immunodeficiency syndrome (AIDS) patients benefit from ART, in resource-limited countries, the proportion of patients who switch their ARTs from first-line to second-line when failing the first-line regimen is increasing. Earlier detection of treatment failure and switching to second-line protease-inhibitor (PI) -based ART probably reduces mortality^3^,^4^,^5^,^6^. LPV/r (a co-formulation of lopinavir and ritonavir) is the first ritonavir-boosted PI and is most widely used as a standard comparator for other boosted PI regimens. Several randomized controlled trials (RCTs) have described clinical outcomes of patients on first-line^7^,^8^,^9^,^10^,^11^,^12^,^13^,^14^ and second-line therapy^15^,^16^,^17^,^18^. Hence, the primary purpose of the present meta-analysis is to evaluate the effectiveness of lopinavir/ritonavir (LPV/r)-based regimens for treatment-naïve HIV-1-infected patients or ART-experienced patient from reported RCTs. PI-based and nucleoside reverse transcriptase inhibitors (NRTI)-containing regimens have also been associated with metabolic perturbations, including hyperlipidemia, insulin resistance, and fat redistribution^7^,^8^,^12^. Considering these metabolic perturbations, another important objective of the study is to evaluate the toxicity related to LPV/r-based ART regimen, focusing on the lipid profile.

Further, it was noticed that there are no adequate studies related to HIV-infected pregnant women. Clinical studies have shown that wherever ART is available widely, it has reduced the mother-to-child transmission (MTCT) rates to 0–3.6%^19^,^20^,^21^,^22^,^23^,^24^,^25^. According to the most recent guidelines from US Department of Health and Human Services, LPV/r is the preferred PI for use in HIV-infected pregnant women^3^. Consequently, along with the growing number of HIV-infected women giving birth, concern has been raised on HIV-1 infection in newborns and the associated birth defects. Hence, considering these facts, the third objective of our study is to evaluate the effects of LPV/r in preventing MTCT of HIV, and also to evaluate its effect on the preterm and low body weight birth rates.

## Results

### General study information

**Figure 1:**
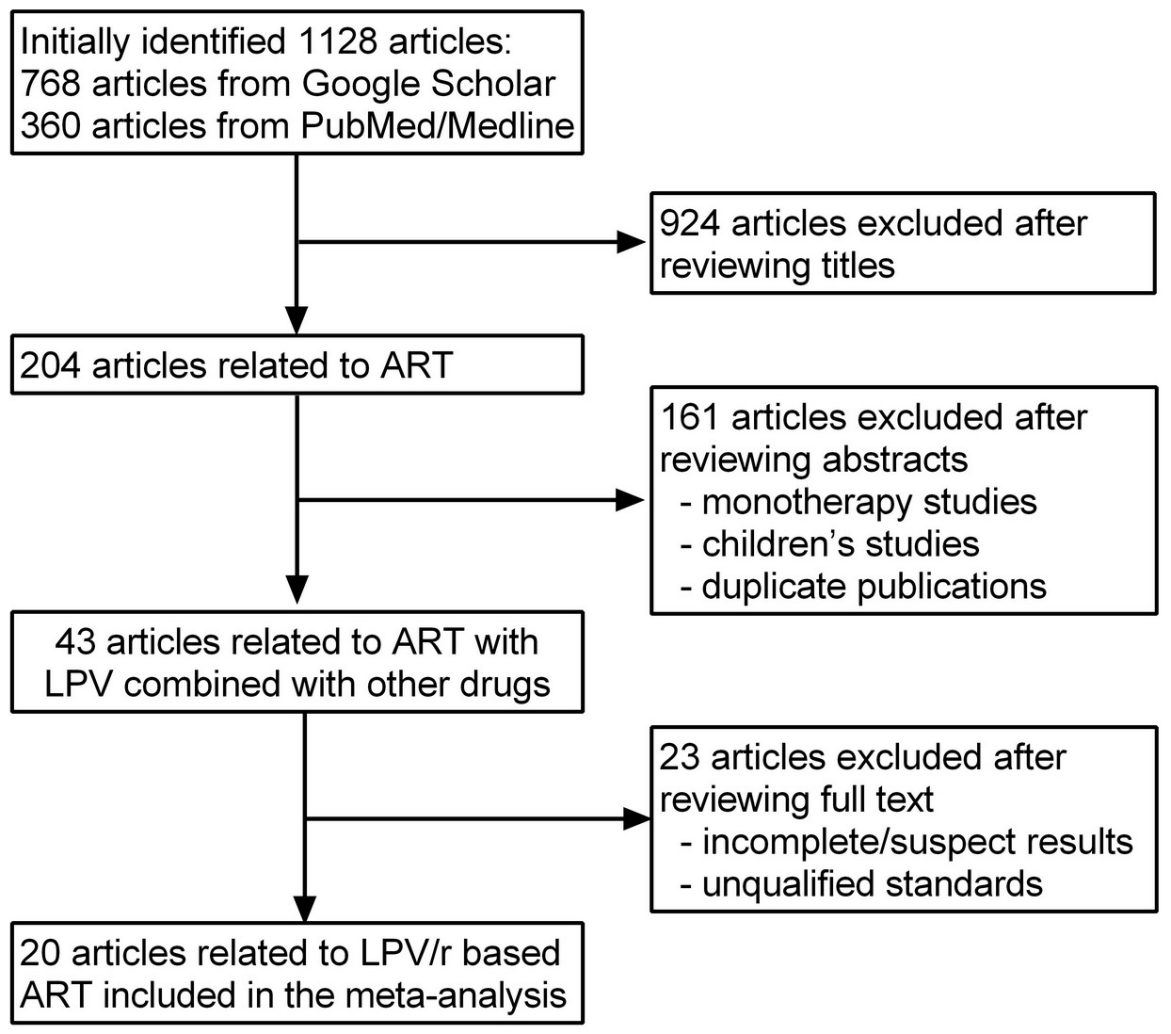
Flow diagram of the study selection process for the meta-analysis.

The search strategy initially identified 1,128 articles in total, of which 768 articles from Google Scholar and 360 articles from PubMed/Medline. Out of the total retrieved articles, the studies excluded after reviewing the titles and abstracts were 924 and 161, respectively. Of the remaining 43 studies that assessed LPV combined with other ART drugs to treat HIV-infected patients, 23 studies were finally excluded from the present study after detailed review for various reasons. Therefore, 8 articles^7^,^8^,^9^,^10^,^11^,^12^,^13^,^14^ involving 2,584 ART-naïve patients, 4 articles^15^,^16^,^17^,^18^ involving 1,124 ART-experienced patients, and 7 articles^19^,^20^,^21^,^22^,^23^,^24^,^25^ involving 2,191 pregnant women were used in the meta-analyses. The detailed process of our literature search is shown in Figure 1. The characteristics of these studies are listed in Table 1.

### Efficacy and biological safety of LPV/r in ART-naïve and -experienced patients

Effectiveness of LPV/r was assessed in 13 studies on 15 cohorts of ART-naïve and -experienced patients. Efficacy was determined on the basis of the virologic response (viral load < 50 copies/mL) rate after 48-weeks' treatment and the changes in VL and CD4^+^ T lymphocyte count. Data related to changes in lipid levels were used to evaluate the safety. According to the information provided in the publications, meta-analyses were conducted on the virologic response of all the cohorts. The changes in CD4^+^ T lymphocyte count and the blood lipid levels were described without meta-analysis due to the lack of enough information. Additionally, although almost all 15 studies in Table 1 have reported the baseline viral load data, only two^18^,^20^ of them reported the viral load data after 48-weeks treatment. Therefore, viral load data were not included in our meta-analysis.

#### Efficacy

**Figure 2:**
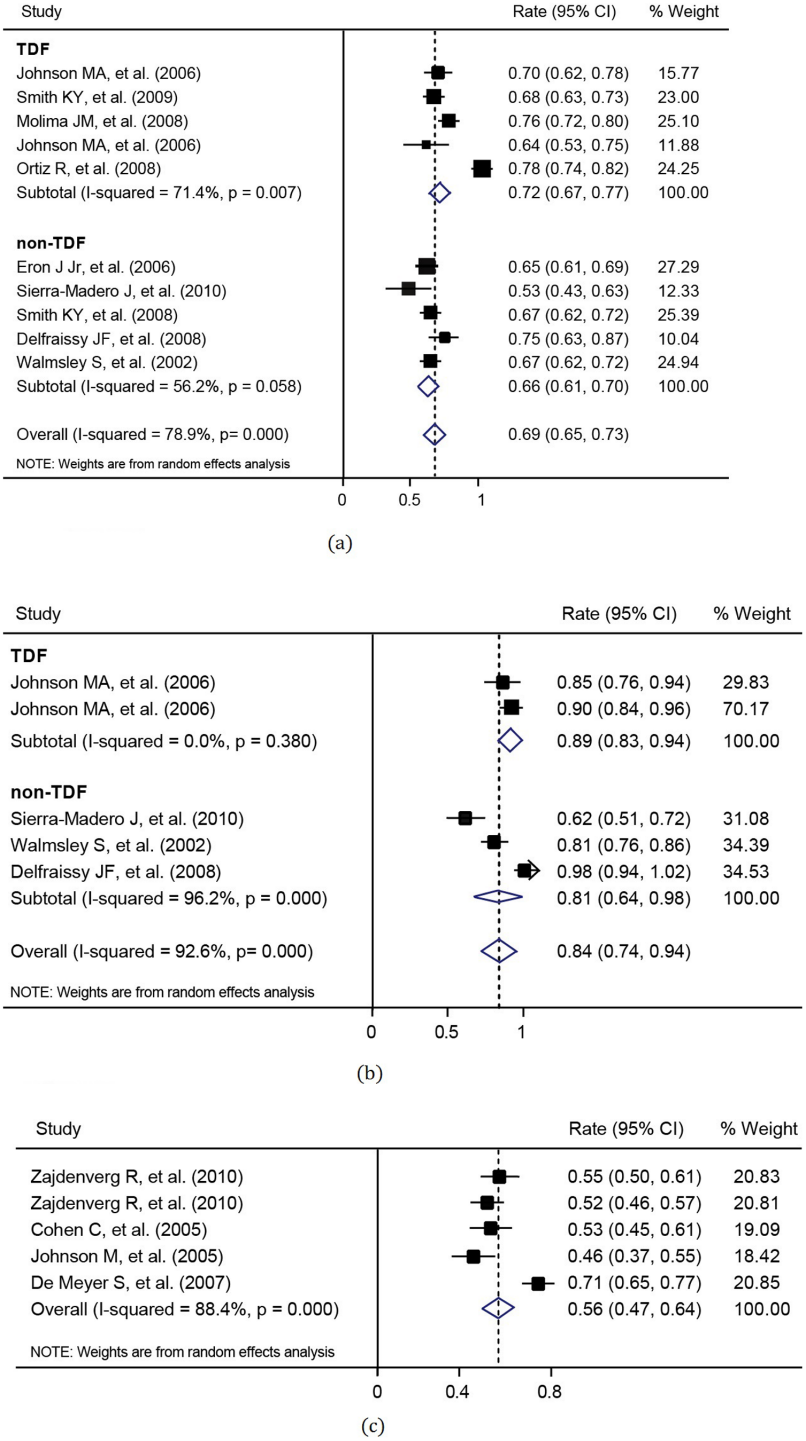
Meta-analysis of virologic response rates for ART-naïve patients under (a) intention-to-treat analysis (heterogeneity: *I*^2^ = 78.9% and p < 0.001; publication bias: p = 0.721); and (b) pre-protocal analysis (heterogeneity: *I*^2^ = 92.6% and p < 0.001; publication bias: p = 0.221).

**Figure 3:**
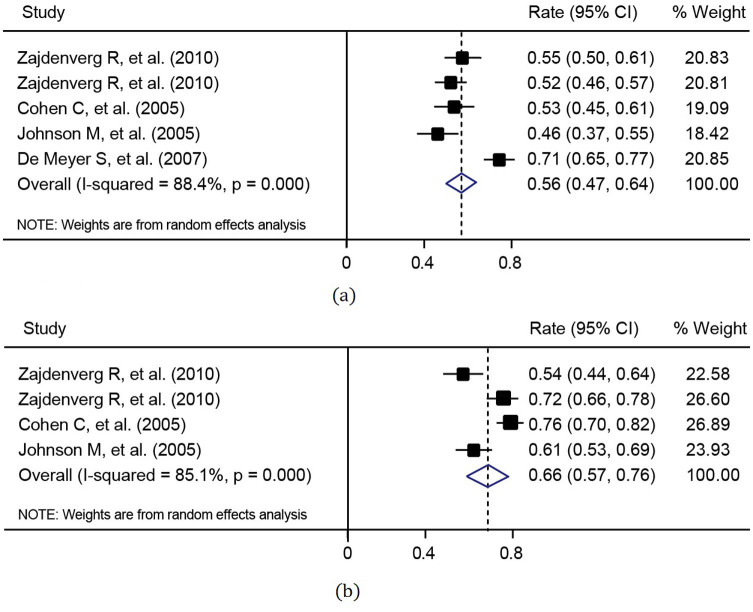
Meta-analysis of virologic response rates for treatment-experienced patients under (a) intention-to-treat analysis (heterogeneity: *I*^2^ = 88.4% and p < 0.001; publication bias: p = 0.086); and (b) pre-protocol analysis (heterogeneity: *I*^2^ = 85.1% and p < 0.001; publication bias: p = 0.089).

For the efficacy measure of virologic response rate, data from 8 articles with 10 cohorts for ART-naïve patients and from 4 articles with 5 cohorts for ART-experienced patients were used in the meta-analysis. The virologic response rates in these studies and the combined virologic response rates are listed in Table 2. It was shown that the virologic response rate of ART-naïve patients with the use of LPV/r was statistically higher than that of ART-experienced patients, using either intention-to-treat (ITT) analysis (*p* = 0.018) or pre-protocol (PP) analysis (*p* = 0.019). Furthermore, ART drugs for ART-naïve patients were LPV/r combined with TDF in 5 cohorts and with non-TDF in the other 5 cohorts, respectively. The combined virologic response rate (72.3%, 95% CI: 67.5–77.1%) of LPV/r plus TDF arms was significantly greater than that of LPV/r plus non-TDF arms (65.5%, 95% CI: 61.2–69.8%, p = 0.047). The meta-analyses were illustrated in Figures 2 and 3. On the other hand, PP analysis showed the combined virologic response rates of 89% and 81% for LPV/TDF and LPV/non-TDF arms, respectively.

The baseline CD4^+^ cell counts and the changes in CD4^+^ cell counts after 48-weeks' treatment from baselines in each study are shown in Table 3.

#### Safety

Table 3 also depicts the assessed fasting blood lipid levels, including directly measured blood lipids values, at baseline and 48-weeks post-therapy. The combined incidence of grade 3 or 4 abnormal total cholesterol (defined as >300 mg/dL) for ART-naïve patients was calculated as 13.1% (95% CI: 11.4–14.8%) using meta-analysis, which was statistically higher than that for ART-experienced patients (6.9%, 95% CI: 4.9–9.0%, p < 0.001). Whereas, the combined incidence of grade 3 or 4 abnormal triglycerides (defined as >750 mg/dL) for ART-naïve patients was 6.0% (95% CI: 4.8–7.1%), which was not statistically different from that for ART-experienced patients (5.5%, 95% CI: 3.7–7.3%, p = 0.363).

### Efficacy of LPV/r in pregnant women

**Figure 4:**
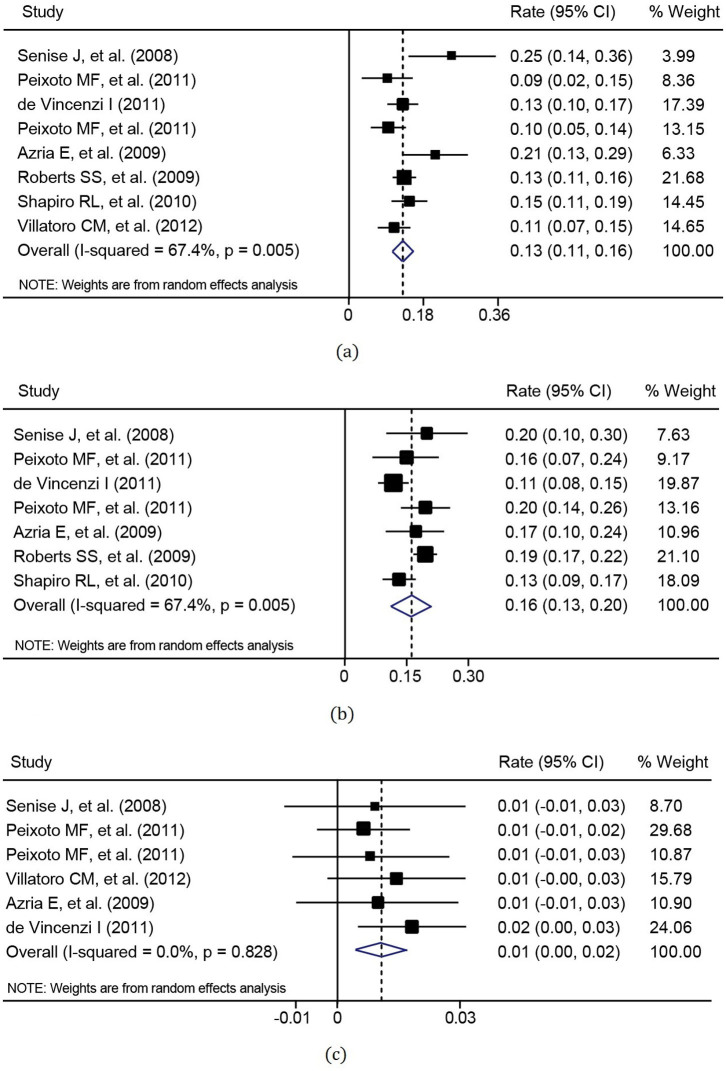
Meta-analysis of the efficacy for pregnant women in terms of (a) preterm birth rate (heterogeneity: *I*^2^ = 51.7% and p = 0.043, publication bias: p = 0.536), (b) low birth weight rate (heterogeneity: *I*^2^ = 67.4% and p = 0.005, publication bias: p = 1.000) and (c) mother-to-child transmission rate of HIV (heterogeneity: *I*^2^ = 0% and p = 0.828, publication bias: p = 0.707).

Seven studies assessed the anti-retroviral effects of LPV/r in 8 cohorts of pregnant women. The assessment included the MTCT rate of HIV and the rates of preterm birth (<37 weeks gestation) and low birth weight (<2500 g). In studies reporting MTCT, preterm delivery and low birth weight, rates ranged from 0.6 to 1.8%, 8.7 to 25% and 11.4 to 20.3%, respectively. Other information about these studies is shown in Table 4. The combined preterm delivery rate, low birth weight rate, and MTCT rate were 13.2% (95% CI: 10.9–15.5%), 16.2% (95% CI: 12.9–19.5%), and 1.1% (95% CI: 0.4–1.7%), respectively (Figure 4).

## Discussion

At present, boosted PIs are the most recommended first-line therapy for NRTI- or non-nucleoside reverse-transcriptase inhibitors (NNRTI)-resistant patients and also the suggested therapy during the planning of pregnancy^3^,^4^,^5^,^6^. LPV/r is the first ritonavir-boosted PI and is most widely used as a standard comparator for other boosted PI regimens. Many RCT studies focused on the assessment of the effectiveness of LPV/r. Though RCTs can provide the highest levels of evidence, single studies still have insufficient statistical power. Therefore, we conducted a meta-analysis to evaluate and describe the efficacy, safety, and tolerability of LPV/r-based ART regimens in a large number of HIV-infected patients. Overall, HIV-infected people on an LPV/r-containing regimen experienced significant virologic and immunologic responses through their first year of therapy.

In the ITT analysis for ART-naïve patients, the proportion of individuals with respect to virologic response rate was slightly different between LPV/r plus TDF and LPV/r plus non-TDF arms (72.3% vs. 65.5%, p = 0.047). However, other studies (one RCT^11^ and two non-RCTs^26^,^27^) comparing ABC/3TC- and TDF/FTC-based therapy with LPV/r in ART-naïve patients suggested no difference (68% vs. 67%, 63% vs. 67%, and 88% vs. 95%, respectively) in virologic response to HIV-1 RNA below 50 copies/mL after receiving therapy for 48 weeks. Our meta-analysis was based on a large number of patients from RCT studies, so the result will be more reliable. In this meta-analysis, the use of LPV/r in HIV-infected subjects with a first-line ART led to virological success in most patients. Even a previous study^9^ showed that 98% of patients reached the virologic response level after receiving therapy for 48 weeks. On the other hand, the use of LPV/r in subjects failing a first-line ART also led to a virological success in more than half (55.7%) of the patients. Therefore, LPV/r played a major role in ushering in the era of boosted PI therapy, and in offering the first good option to patients who had failed prior therapy.

In addition, our results indicated that LPV/r can effectively improve the immunological outcome. After treatment for 48 weeks, CD4^+^ counts increased to 141–239 cells/mm^3^ from baseline. In the CASTLE study^12^, a prospective, open-label, randomized study to determine the safety and efficacy of atazanavir/ritonavir compared to LPV/r, CD4^+^ counts increased to 219 cells/mm^3^ from baseline. Even for patients with severely impaired baseline immune function, in whom the initial median level of CD4^+^ count was only 54 cells/mm^3^, LPV/r showed significant immunological efficacy by boosting CD4^+^ counts to 239 cells/mm^3^ from baseline to week 48^14^. More importantly, ART-experienced patients showed remarkable immunological efficacy with elevated CD4^+^ counts of 121–169 cells/mm^3^ 15,16,17. Hence, even in the ART-experienced patients, LPV/r still showed robust efficacy to elevate CD4^+^ counts with few virological failures.

Grade 3 or 4 treatment-related hyperlipemia was reported in ART patients. In 2 studies carried out by Ortiz *et al*.^8^ and Molina *et al*.^12^, the incidence of dyslipidemia in ART-naïve patients was 23% and 18%, respectively. Similarly, a greater risk of hypertriglyceridemia was found in ART-naïve patients in our analysis. We are encouraged that these data demonstrate that LPV/r was well tolerated in ART-experienced patients in terms of lipid levels, as the incidence of abnormal total cholesterol in ART-experienced patients was 6.9%, which was 13.1% in ART-naïve patients (p < 0.001). Other drugs that could potentially influence triglyceride levels were well balanced between at baseline and during follow-up. However, in the meta-analysis, the number of individuals with available low-density lipoprotein measurements was lower than with the two other lipid factors, especially in the ART-experienced arm; nevertheless, in a clinical setting our results confirmed that LPV/r is a valid option which presents a good tolerability among the ART-experienced patients. Therefore, LPV/r is used as the second-line regimen with good tolerated in China. Consequently, it is highly recommended that lipid levels are measured before commencement of therapy and should be monitored periodically during follow-up.

Different studies have provided different answers to the question of whether the use of LPV/r-based ART during pregnancy confers an increased risk of preterm delivery. The meta-analysis of eight cohorts of 2,191 pregnant women with LPV/r resulted in a relatively low MTCT rate of 1.1%, preterm birth rate of 13.2%, and a low birth weight rate of 16.2%, which were similar to those of HIV-negative women in a small prospective cohort of six US centers^28^. However, it was lower than previously reported values which showed a prematurity rate of 19.1% in ART-treated HIV-infected women^29^,^30^,^31^. These meta-analysis findings showed that ART regimens currently being used to treat HIV-infected women during pregnancy are not associated with an increased risk of premature delivery and low birth weight. Therefore, it can be said that the LPV/r regimen is a relatively safe treatment option in terms of newborn health. The results from another systematic review^32^ further suggested that there were no unique safety or efficacy concerns with the use of standard dose LPV/r as part of ART regimens in pregnant women.

A limitation of this study is that it only included publications in English. Moreover, for the meta-analysis of the efficacy of LPV/r for HIV-infected pregnant women, observational studies are prone to bias because the groups compared may be dissimilar in characteristics. Factors including local medical environments, maternal race, age, and previous obstetric history other than treatment might also be responsible for premature birth. It is also possible that different ART classes of agents, or even agents within each class, inconsistent research durations, and different time points of therapy might have different effects on the risk of premature delivery.

This study demonstrated sufficient evidence to show that LPV/r was an efficacious regimen for ART-naïve patients and was more tolerable for ART-experienced patients. In addition, LPV/r displayed a significant effect in preventing MTCT.

## Methods

### Strategy for literature search

A computer-based literature search was conducted using search engines including Google Scholar and PubMed/Medline with ‘lopinavir/ritonavir' and ‘HIV/AIDS' as the search terms in the titles. Subsequently, literature on ART using LPV/r combined with other drugs was collected.

### Study selection

Studies that assessed the effectiveness of LPV/r-based ART in HIV-infected patients, recruited adult HIV-infected patients, and gave the efficacy and/or safety outcomes were included in the current meta-analysis. Whereas the reviews of LPV/r treatment in HIV, pharmacokinetic studies of LPV/r in HIV-infected patients, studies that recruited HIV-infected children, duplicate publications or studies with similar data collection, and studies with incomplete data were excluded.

Study selection was performed by reviewing the titles and abstracts of all examined articles, followed by a detailed review of the eligible articles. This process was carried out independently by 2 researchers (Q. Y. Yang and T. Zhang) without prior consideration of the results. They came to a consensus through discussion after any disagreement.

### Data extraction

Two researcher (J. Q. Chen and Y. L. Xu) independently extracted and then cross-checked the following data: the name of the first author, study design, publication year, the number of patients, analytic method, patients' characteristics (whether received ART and ART drugs used), baseline information of each patient (viral load, CD4^+^ T cell count, total cholesterol, triglyceride and low-density lipoprotein), and relevant outcome data. For HIV-infected adults, the primary outcomes of efficacy and safety were the virologic response rate and fasting lipid levels (including directly measured blood lipids values), respectively. For pregnant women, the primary efficacy outcome was the mother-to-child transmission rate. Any disagreements were resolved by discussion and consensus.

### Statistical analysis

Statistical analyses were conducted by Stata, version 12 (StataCorp LP, USA). Heterogeneity for each combined rate was assessed by chi-square-based Q-test and the *I*^2^ statistic. Heterogeneity was considered as moderate to large when *P* < 0.1 for Q-test or *I*^2^ < 50%. Meta-analyses were conducted via random effects models for studies presenting moderate to large heterogeneity, otherwise, fixed effects models were used. Publication bias was evaluated by Begg's test. Two combined rates were compared by the method described in Altman's article^33^.

## Author Contributions

X.H. and H.W. wrote the main manuscript text, Q.Y., J.C., T.Z. and Y.X. searched the library and reviewed all articles, J.C. and H.C. conducted all meta-analysis, Z.L. and C.G. prepared all figures, N.L. wrote part of the manuscript. All authors reviewed the manuscript.

## Figures and Tables

**Table 1:** General information of studies included in the meta-analysis

Authors	Publication year	Number of patients	Drug combination†	Analytic method	Study design
ART-naïve patients					
Walmsley S *et al*.^7^	2002	326	d4T-3TC + LPV/r/Nelfinavir	ITT/PP	RCT
Ortiz R *et al*.^8^	2008	346	TDF-FTC + LPV/r/darunavir/r	ITT	RCT
Delfraissy JF *et al*.^9^	2008	53	AZT-3TC + LPV/r	ITT/PP	RCT
Johnson MA *et al*.^10^	2006	115	TDF-FTC + LPV/r	ITT/PP	RCT
Johnson MA *et al*.^10^	2006	75	TDF-FTC + LPV/r	ITT/PP	RCT
Smith KY *et al*.^11^	2009	345	TDF-FTC + LPV/r	ITT	RCT
Smith KY *et al*.^11^	2009	343	ABC-3TC + LPV/r	ITT	RCT
Molina JM *et al*.^12^	2008	443	TDF-FTC + LPV/r/atazanavir/r	ITT	RCT
Eron J Jr *et al*.^13^	2006	444	ABC-3TC + LPV/r/fosamprenavir-r	ITT	RCT
Sierra-Madero J *et al*.^14^	2010	94	AZT-3TC + LPV/r/EFV	ITT/PP	RCT
ART-experienced patients					
Cohen C *et al*.^15^	2005	150	AZT-3TC/d4T-3TC/AZT-DDI/d4T-DDI + LPV/r	ITT/PP	RCT
Zajdenverg R *et al*.^16^	2010	300	≥2 NRTIs (AZT/3TC/ABC/DDI/d4T/TDF/FTC) + LPV/r	ITT/PP	RCT
Zajdenverg R *et al*.^16^	2010	299	≥2 NRTIs (AZT/3TC/ABC/DDI/d4T/TDF/FTC) + LPV/r	ITT/PP	RCT
Johnson M *et al*.^17^	2005	123	TDF + one NRTI(DDI/d4T/3TC/AZT/ABC)+ LPV/r	ITT/PP	RCT
De Meyer S *et al*.^18^	2007	252	(NRTI + one NNRTI) + LPV/r	ITT	RCT
Pregnant women					
Roberts SS *et al*.^19^	2009	890	LPV/r	ITT	Prospective
Senise J *et al*.^20^	2008	64	AZT-3TC + LPV/r	ITT	Retrospective
de Vincenzi I^21^	2011	401	AZT-3TC + LPV/r	ITT	RCT
Azria E *et al*.^22^	2009	100	AZT-3TC/AZT-other NRTI/AZT-alone+ LPV/r	ITT	Retrospective
Peixoto MF *et al*.^23^	2011	164	LPV/r	ITT	Prospective
Peixoto MF *et al*.^23^	2011	70	LPV/r	ITT	Prospective
Villatoro CM *et al*.^24^	2012	219	LPV/r alone or AZT-3TC + LPV/r	ITT	Retrospective
Shapiro RL *et al*.^25^	2010	283	AZT-3TC/ABC-3TC + LPV/r	ITT	Prospective

ITT: intention-to-treat; PP: per-protocol; RCT: randomized controlled trial.

There were two different dose groups of ART-experienced patients with LPV/r tablets 800/200 mg QD (n = 300) or 400/100 mg BID (n = 299) in one study conducted by Zajdenverg R et al.

^†^LPV/r in combination with an optimized background regimen of at least 2 nucleoside/nucleotide reverse transcriptase inhibitors (NRTIs). The most commonly used drugs are TDF/AZT/D4T/ABC,DDI,3TC/FTC.

**Table 2:** Virologic response rates for ART-naïve and -experienced patients using intention-to-treat analysis and pre-protocol analysis

	Using ITT analysis		Using PP analysis	
	Range	Combined rate (95% CI)	Range	Combined rate
ART-naïve patients	53–78%	68.8% (64.8–72.9%) †	62–98%	83.8% (73.6–94.0%)‡
ART-experienced patients	46–71%	55.7% (47.1–64.2%)	54–76%	66.4% (57.3–75.6%)

ITT: intention-to-treat; PP: per-protocol; CI: confidence interval.

^†^Compared to ART-experienced patients, p = 0.018;

^‡^Compared to ART-experienced patients, p = 0.019.

**Table 3:** Changes in CD4^+^ count and blood lipid levels (48-weeks post-treatment)

	Total cholesterol (mg/dL)			Triglycerides (mg/dL)			Low-density lipoprotein (mg/dL)			CD4^+^ count(cells/mm^3^)	
Study	Baseline	Week 48	Abnormal rate (%)#	Baseline	Week 48	Abnormal rate (%)$	Baseline	Week 48	Abnormal rate (%)∧	Baseline	ΔCD4^+^
ART-naïve patients											
Walmsley S *et al*.^7^	NA	53†	9	NA	125†	9.3	NA	NA		260	207
Ortiz R *et al*.^8^	NA	NA	23	NA	NA	11	NA	NA	10	218	141
Johnson MA *et al*.^10^	159	27†	NA	137	82†	5	96	14†	NA	214 (116–380) ‡	185
Johnson MA *et al*.^10^	168	27†	NA	136	76†	4	102	13†	NA	232 (95–339)‡	188
Molina JM *et al*.^12^	147	185	18	110	168	4	91	108	NA	204	219
Eron J Jr *et al*.^13^	157	210	9	117	195	8	97	120	NA	194 (79–287)‡	191 (124–287)‡
Sierra-Madero J *et al*.^14^	NA	63†	NA	NA	116†	NA	NA	10†	NA	52 (37.1–66.8)‡	239
ART-experienced patients											
Cohen C *et al*.^15^	167	190	NA	162	211	NA	97	103	NA	256	169
Zajdenverg R *et al*.^16^	NA	NA	6.5	NA	NA	4.8	NA	NA	NA	239.3	153
Zajdenverg R *et al*.^16^	NA	NA	7.5	NA	NA	6.4	NA	NA	NA	268.3	122

^†^Elevated value;

^#^Grade 3 or 4 abnormal: defined as >300 mg/dL except for Molina's study (Ref. 12);

^$^Grade 3 or 4 abnormal: defined as >750 mg/dL;

^∧^Grade 3 or 4 abnormal was not mentioned;

^‡^median (quartiles).

**Table 4:** General information about LPV/r treatment of pregnant women

	Age at delivery (year)	Baseline CD4^+^ count (cells/mm^3^)	Baseline viral load (log10 copies/mL)	Vaginal delivery (%)	MTCT (%)	PD (%)	LBW (%)
Roberts SS *et al.*^19^	13–48	NA	NA	NA	NA	13.4	19.2
Senise J *et al*.^20^	29.4 (16–41)#	289 (13–811)*	4.28 (0– ≥ 5.88)*	11	0.8	25.0	20.3
de Vincenzi I^21^	27 (24–31)*	336 (282–408)∧	4.23 (3.66–4.75)∧	89	1.8	13.2	11.4
Azria E *et al*.^22^	32.4 ± 5.0$	361 (8–858)*	3.6 (<1.7–5.4)*	45	1.0	21.0	17.0
Peixoto MF *et al*.^23^	29.6 ± 5.5$	486.1 ± 292.7 $	2.6 ± 1.0$	NA	0.6	9.8	20.2
Peixoto MF *et al*.^23^	27.1 ± 6.4$	535.4 ± 303.9 $	3.0 ± 0.7$	NA	0.7	8.7	15.9
Villatoro CM *et al*.^24^	26 (16–43)*	329 (2–1034)#	4.82 (0–6.26)#	4	1.4	10.6	NA
Shapiro RL *et al*.^25^	25	403 (297–514)∧	3.96 (3.34–4.60)∧	NA	NA	14.8	13.1

^#^Mean (range);

*median (range);

^∧^median (quartiles);

^$^mean ± standard deviation.

MTCT: mother-to-child transmission; PD: preterm delivery (<37 weeks gestation); LBW: low birth weight (<2,500 g).

## References

[b1] Palella F. J. Jr *et al.* Declining morbidity and mortality among patients with advanced human immunodeficiency virus infection. N Engl J Med 338, 853–860 (1998). 9516219 10.1056/NEJM199803263381301

[b2] Schackman B. R. *et al.* The lifetime cost of current human immunodeficiency virus care in the United States. Med Care 44, 990–997 (2006). 17063130 10.1097/01.mlr.0000228021.89490.2a

[b3] Panel on Antiretroviral Guidelines for Adults and Adolescents. Guidelines for the use of antiretroviral agents in HIV-1-infected adults and adolescents. Department of Health and Human Services. Available at: http://aidsinfo.nih.gov/guidelines. (Accessed 30th August 2014)

[b4] Thompson M. A. *et al.* Antiretroviral treatment of adult HIV infection: 2012 recommendations of the International Antiviral Society-USA panel. JAMA 308, 387–402 (2012). 22820792 10.1001/jama.2012.7961

[b5] Williams I. *et al.* British HIV Association guidelines for the treatment of HIV-1-positive adults with antiretroviral therapy 2012 (Updated November 2013). HIV Med 15 Suppl 1,1–85 (2014). 10.1111/hiv.12119 24330011

[b6] World Health Organization. Antiretroviral therapy for HIV infection in adults and adolescents: recommendations for a public health approach (2006 revision.). Available at: http://www.who.int/hiv/pub/guidelines/. (Accessed 30th August 2014) 23741771

[b7] Walmsley S. *et al.* Lopinavir-ritonavir versus nelfinavir for the initial treatment of HIV infection. N Engl J Med 346, 2039–2046 (2002). 12087139 10.1056/NEJMoa012354

[b8] Ortiz R. *et al.* Efficacy and safety of once-daily darunavir/ritonavir versus lopinavir/ritonavir in treatment-naive HIV-1-infected patients at week 48. AIDS 22, 1389–1397 (2008). 18614861 10.1097/QAD.0b013e32830285fb

[b9] Delfraissy J. F. *et al.* Lopinavir/ritonavir monotherapy or plus zidovudine and lamivudine in antiretroviral-naive HIV-infected patients. AIDS 22, 385–393 (2008). 18195565 10.1097/QAD.0b013e3282f3f16d

[b10] Johnson M. A. *et al.* A once-daily lopinavir/ritonavir-based regimen provides noninferior antiviral activity compared with a twice-daily regimen. JAIDS - J Acq Imm Def Syndr 43, 153–160 (2006). 10.1097/01.qai.0000242449.67155.1a 16951643

[b11] Smith K. Y. *et al.* Randomized, double-blind, placebo-matched, multicenter trial of abacavir/lamivudine or tenofovir/emtricitabine with lopinavir/ritonavir for initial HIV treatment. AIDS 23, 1547–1556 (2009). 19542866 10.1097/QAD.0b013e32832cbcc2

[b12] Molina J. M. *et al.* Once-daily atazanavir/ritonavir versus twice-daily lopinavir/ritonavir, each in combination with tenofovir and emtricitabine, for management of antiretroviral-naive HIV-1-infected patients: 48 week efficacy and safety results of the CASTLE study. Lancet 372, 646–655 (2008). 18722869 10.1016/S0140-6736(08)61081-8

[b13] Eron J. Jr *et al.* The KLEAN study of fosamprenavir-ritonavir versus lopinavir-ritonavir, each in combination with abacavir-lamivudine, for initial treatment of HIV infection over 48 weeks: a randomized non-inferiority trial. Lancet 368, 476–482 (2006). 16890834 10.1016/S0140-6736(06)69155-1

[b14] Sierra-Madero J. *et al.* Prospective, randomized, open label trial of efavirenz vs. lopinavir/ritonavir in HIV+ treatment-naive subjects with CD4+200 cell/mm3 in Mexico. JAIDS - J Acq Imm Def Syndr 53, 582–588 (2010). 10.1097/QAI.0b013e3181cae4a1 20090545

[b15] Cohen C. *et al.* Comparison of atazanavir with lopinavir/ritonavir in patients with prior protease inhibitor failure: a randomized multinational trial. Curr Med Res Opin 21, 1683–1692 (2005). 16238909 10.1185/030079905x65439

[b16] Zajdenverg R. *et al.* Similar safety and efficacy of once- and twice-daily lopinavir/ritonavir tablets in treatment-experienced HIV-1-infected subjects at 48 weeks. JAIDS - J Acq Imm Def Syndr 54, 143–151 (2010). 10.1097/QAI.0b013e3181cbd21e 20134330

[b17] Johnson M. *et al.* Atazanavir plus ritonavir or saquinavir, and lopinavir/ritonavir in patients experiencing multiple virological failures. AIDS 19, 685–694 (2005). 15821394 10.1097/01.aids.0000166091.39317.99

[b18] De Meyer S. *et al.* Effect of baseline and on-treatment mutations on the antiretroviral activity of darunavir/ritonavir and lopinavir/ritonavir: results of a randomized, controlled, phase III study (TITAN). 4th International AIDS Society Conference on HIV Pathogenesis, Treatment and Prevention. Sydney 2007. Abstract WEPEB038.

[b19] Roberts S. S. *et al.* Lopinavir/ritonavir in pregnancy. JAIDS - J Acq Imm Def Syndr 51, 456–461 (2009). 10.1097/QAI.0b013e3181a2813f 19381099

[b20] Senise J. *et al.* Low-birth weight and pre-term delivery in relation to lopinavir/ritonavir use in pregnancy. J Am Infect Dis 4, 209–214 (2008).

[b21] de Vincenzi I. Triple antiretroviral compared with zidovudine and single-dose nevirapine prophylaxis during pregnancy and breastfeeding for prevention of mother-to-child transmission of HIV-1 (Kesho Bora study): a randomized controlled trial. Lancet Infect Dis 11, 171–180 (2011). 21237718 10.1016/S1473-3099(10)70288-7

[b22] Azria E. *et al.* Pregnancy outcomes in women with HIV type-1 receiving a lopinavir/ritonavir-containing regimen. Antiviral Ther 14, 423–432 (2009). 19474476

[b23] Peixoto M. F. *et al.* Lopinavir/ritonavir dosing during pregnancy in Brazil and maternal/infant laboratory abnormalities. Braz J Infect Dis 15, 253–261 (2011). 21670927 10.1016/s1413-8670(11)70185-4

[b24] Villatoro C. M. *et al.* Highly Active Antiretroviral Treatment (HAART) for the Prevention of HIV Mother to Child Transmission (PMTCT) at Roosevelt Hospital's Infectious Diseases Clinic in Guatemala: The Role of (LPV/r) Standard Dose. World J AIDS 2, 259–264 (2012).

[b25] Shapiro R. L. *et al.* Antiretroviral regimens in pregnancy and breast-feeding in Botswana. N Engl J Med 362, 2282–2294 (2010). 20554983 10.1056/NEJMoa0907736 PMC2999916

[b26] Wolf E. *et al.* Similar virological response rates for ART-naive subjects starting KVX + LPV/r or TVD + LPV/r: Data from the prospective observational STAR cohort. J Int AIDS Society 11 (Suppl 1), 7 (2008).

[b27] Eccleston K. J., Bambumba A., Babu C. S., Ahmed S. & Lee V. Efficacy and safety of tenofovir/emtricitabine compared to abacavir/lamivudine in HIV-1 infected patients in clinical setting: the TEAL study. J Int AIDS Society 11 (Suppl 1), 79 (2008).

[b28] Massad L. S. *et al.* Pregnancy rates and predictors of conception, miscarriage and abortion in US women with HIV. AIDS 18, 281–286 (2004). 15075546 10.1097/00002030-200401230-00018

[b29] Morris A. B. *et al.* Multicenter review of protease inhibitors in 89 pregnancies. JAIDS-J Acq Imm Def Syndr 25, 306–311 (2000). 10.1097/00042560-200012010-00003 11114830

[b30] Centers for Disease Control and Prevention (CDC). Preterm singleton births – United States, 1989-1996. MMWR Morb Mortal Wkly Rep 48, 185–189 (1999). 10208123

[b31] Kourtis A. P. *et al.* Hospitalizations of pregnant HIV-infected women in the USA prior to and during the era of HAART, 1994–2003. AIDS 20, 1823–1831 (2006). 16954723 10.1097/01.aids.0000244201.11006.1c

[b32] Pasley M. V., Martinez M., Hermes A., d'Amico R. & Nilius A. Safety and efficacy of lopinavir/ritonavir during pregnancy: a systematic review. AIDS Rev 15, 38–48 (2013). 23449228

[b33] Altman D. G. & Bland J. M. Statistics Notes: Interaction revisited: the difference between two estimates. Bri Med J 326, 219 (2003). 10.1136/bmj.326.7382.219 PMC1125071 12543843

